# Two New Flavonol Glycosides from *Polygala sibirica* L. var *megalopha* Fr.

**DOI:** 10.3390/molecules201219775

**Published:** 2015-12-02

**Authors:** Yan-Jie Huang, Ling-Yun Zhou, Jun-Min Wang, Qiang Li, Yuan-Yuan Geng, Hai-Yang Liu, Yan Hua

**Affiliations:** 1 College of Forestry, Southwest Forestry University, Kunming 650224, Yunnan, China; hyj0106@foxmail.com (Y.-J.H.); zly324@foxmail.com (L.-Y.Z); dawn723@163.com (J.-M.W.); kib312270163@foxmail.com (Q.L.); 18208826431@163.com (Y.-Y.G.); 2 School of Pharmacy, Wannan Medical College, Wuhu 241002, Anhui, China; 3 State Key Laboratory of Phytochemistry and Plant Resources in West China, Kunming Institute of Botany, Chinese Academy of Sciences, Kunming 650204, Yunnan, China

**Keywords:** *Polygala sibirica*, Polygalaceae, flavonol glycosides, inhibition of xanthine oxidase

## Abstract

Two new flavonol glycosides, named polygalin H (**1**) and polygalin I (**2**), as well as the known compound polygalin D (**3**), were isolated from the whole plant of *Polygala sibirica* L. var *megalopha* Fr. Their structures were elucidated on the basis of spectroscopic data analysis. These flavonol glycosides exhibited strong inhibitory activities against xanthine oxidase *in vitro*. Their half-maximal inhibitory concentrations (IC_50_) were calculated, which were 9.48, 8.31, 16.00 μM, respectively.

## 1. Introduction

*Polygala sibirica* L. var *megalopha* Fr. (Polygalaceae), a native medicinal plant of Yunnan Province (China), has long been used in folk medicine for the treatment of fever, inflammation, arthralgia and viper bites [1,2,3,4]. So far, it was reported that polysaccharides and some novel flavonol and xanthone derivatives had been isolated from this plant [5,6], and some reports on high inhibitory activities against xanthine oxidase by these flavonoids and xanthone derivatives were revealed recently [7,8,9]. All of this attracted us to further investigate its chemical constituents and seek more compounds as potential xanthine oxidase inhibitors. In the present work, two new compounds, named as polygalin H (**1**) and polygalin I (**2**), and one known compound polygalin D (**3**) [10,11], were isolated. In this paper, we describe the isolation, structural elucidation of the two new compounds, as well as the inhibition of xanthine oxidase by the three compounds.

## 2. Results and Discussion

**Figure 1:**
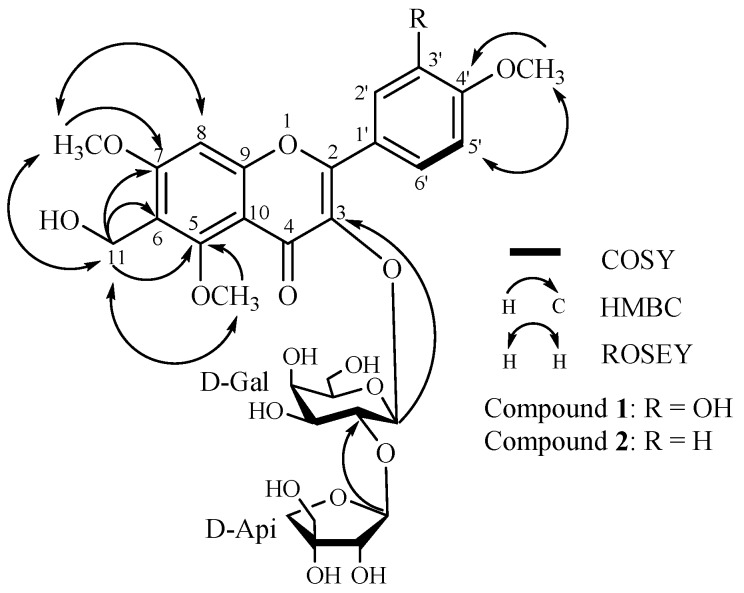
Key COSY, HMBC and ROESY correlations of polygalin H (**1**) and polygalin I (**2**).

Compound **1** was obtained as a yellow amorphous powder. Its molecular formula was determined to be C_30_H_36_O_17_ by MALDI-TOF/TOF-MS (*m*/*z* 691.1077, calcd. for 691.1640, [M + Na]^+^). The compound exhibited IR absorption bands at 3440 and 1613 cm^−1^ and UV maximum absorptions (209, 258, 273 and 343 nm), characteristic of a flavonoid. Besides the eleven sugar signals in the ^13^C-NMR spectrum, the remaining peaks corresponded to signals of three methoxyl [δ_C_ 57.3 (q), 56.5 (q), 55.6 (q)], one oxygenated methylene (δ_C_ 60.8, t), four methines and eleven quaternary carbons. In contrast to polygalin C [11], compound **1** has one additional methoxy signal and another oxygenated methylene signal. The oxygenated methylene moiety was deduced to be linked at C-6 by the HMBC correlations between the oxygenated methylene protons H-11 (δ_H_ 4.39, s, 2H) and C-6 (δ_C_ 108.0), C-5 (δ_C_ 159.3), C-7 (δ_C_ 163.9), which was further confirmed by the evidence that the chemical shift of C-6 (δ_C_ 108.0) in compound **1** moved 10.2 ppm downfield compared with polygalin C. Simultaneously, in the HMBC spectrum, the long-range correlations between the three methoxyl (δ_H_ 3.21, 3.91, 3.87) and their corresponding carbons C-5 (δ_C_ 159.3), C-7 (δ_C_ 163.9), C-4′ (δ_C_ 150.2), indicated the positions of the methoxy moieties. Therefore, the additional methoxy moiety compared with polygalin C was determined to be linked at C-5. These assignments were further confirmed by the ROSEY spectrum (see Figure 1).

Acid hydrolysis of **1** with 1 M HCl afforded two monosaccharides, which were determined to be d-galactopyranose and d-apiofuranose by TLC and GC analysis. In the HMBC spectrum, the long-range correlations indicated that H-1″ (δ_H_ 5.62, d, *J* = 7.7 Hz) of the galactose was attached to C-3 (δ_C_ 133.9) of the aglycone and the H-1‴ (δ_H_ 5.31, s) of the apiose was linked at C-2″ (δ_C_ 74.9) of the galactose. In addition, the coupling constant (*J* = 7.7 Hz) of the anomeric proton in galactose revealed its β-orientation, while the apiose was also deduced to be in an β-orientation by comparing its ^13^C-NMR spectral data (δ_C_ 108.8, 76.1, 79.2, 73.9, 64.2) with literature data [12,13]. Thus, compound **1** was elucidated as 3,3′-dihydroxy-6-hydroxymethyl-5,7,4′-trimethoxyflavone-3-*O*-β-d-apiofuranosyl-(1→2)-β-d-galactopyranoside (see Figure 1), and was named polygalin H.

Compound **2** was also obtained as a yellow amorphous powder. The MALDI-TOF/TOF-MS provided a molecular formula of C_30_H_36_O_16_ at *m*/*z* 675.1942 [M + Na]^+^ (calcd. for 675.1901). The compound showed IR absorption bands at 3441 and 1609 cm^−1^ and UV maximum absorptions (209, 258, 273 and 343 nm). Comparison of the ^13^C-NMR and ^1^H-NMR spectral data of compound **2** with those of compound **1**, showed that the two compounds had similar structures except for one additional proton signal (δ_H_ 7.03, H-3′) in compound **1**. Moreover, the protons of H-2′, and H-6′ (δ_H_ 8.27, d, *J* = 8.5 Hz), H-3′ and H-5′ (δ_H_ 7.03, d, *J* = 8.5 Hz) in compound **2** exhibited a set of AA′BB′-system signals, which indicated that the hydroxyl moiety at C-3′ in compound **1** was replaced by a hydrogen (δ_H_ 7.03, d, *J* = 8.5 Hz) in compound **2**. Thus, the compound **2** was elucidated to be 3-hydroxy-6-hydroxymethyl-5,7,4′-trimethoxyflavone-3-*O*-β-d-apiofuranosyl-(1→2)-β-d-galactopyranoside (see Figure 1), and named polygalin I.

**Figure 2:**
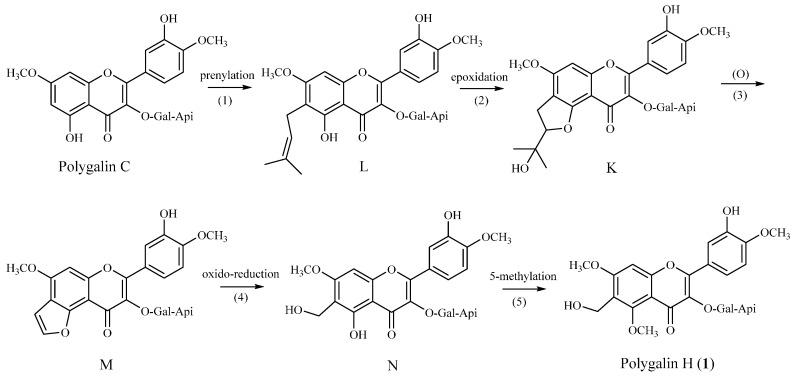
Hypothetical biosynthesis of polygalin H (**1**).

It is noteworthy that the hydroxymethyl function in the skeletons of these flavonoids is reported for the first time. Based on the furocoumarin biosynthetic pathway [14], we put forward a hypothesis that polygalin H (**1**) is derived from polygalin C, which we also had obtained from *P. Sibirica* L. Var *megalopha* Fr. previously. In our hypothesis (see Figure 2), we speculate that compounds K and M were unstable, as they were never found among flavones [15,16,17], and the ultimate product, polygalin H, would be obtained through methylation after L was transformed into N. In our following work, the biosynthetic pathway, the chemical properties and bioactivities of flavonoids with this substitution pattern will be further investigated.

Four concentrations (2.5, 5.0, 10.0, 20.0 μM) of the flavonol glycosides were analyzed for inhibition of xanthine oxidase, and then the corresponding half-maximal inhibitory concentration (IC_50_) value was calculated (see Table 1). The results showed that their IC_50_s were 9.48, 8.31, 16.00 μM, respectively, which were a little higher than the IC_50_ value (4.34 μM) of the positive control, allopurinol. Consequently, this indicated that those three flavonol glycosides had strong inhibitory activities against xanthine oxidase.

**Table 1:** IC_50_ values of three flavonol glycosides for inhibition of xantine oxidase.

Compound	Final Concentration (μM)	Inhibition (%)	IC_50_ (μM)
**1**	20.0	76.87 ± 2.0	9.48
	10.0	43.61 ± 2.7	
	5.0	30.71 ± 0.8	
	2.5	15.53 ± 3.8	
**2**	20.0	80.87 ± 4.6	8.31
	10.0	46.35 ± 6.6	
	5.0	33.73 ± 6.1	
	2.5	18.45 ± 5.4	
**3**	20.0	58.79 ± 1.6	16.00
	10.0	33.81 ± 2.7	
	5.0	23.74 ± 1.2	
	2.5	14.12 ± 0.6	
Allopurinol (positive control)	20.0	96.87 ± 1.2	4.34
	10.0	70.61 ± 4.0	
	5.0	45.71 ± 0.8	
	2.5	37.53 ± 3.8	

## 3. Experimental Section

### 3.1. General Information

Optical rotations were recorded using a P-1020 digital polarimeter (Jasco, Tokyo, Japan). The UV spectra were measured on a UV-2401PC spectrophotometer (Shimadzu, Suzhou, China). The IR spectra were recorded as KBr pellets on a Tensor-27 spectrometer (Bruker, Bremen, Germany). The NMR spectra were recorded on an AM-400 spectrometer (Bruker) with TMS as an internal standard. ESI-MS and HR-EI-M-TOF-MS were measured on a Bruker HTC/Esquire spectrometer and a Bruker Daltonics Flex spectrometer, respectively. GC analysis was carried out on an HP-5890 II system (Gentech Scientific, Alto, CA, USA) equipped with a FID detector and a HP-20M capillary column (25 m × 0.32 mm × 0.3 μm). Column chromatography was performed with silica gel (Qingdao Marine Chemical Industry Factory, Qingdao, China) and Sephadex LH-20 (GE Healthcare Bio-Sciences AB, Fairfield, CT, USA) and reversed-phase C18 silica gel (40–60 μm, Merck, Darmstadt, Germany). TLC was performed with silica gel GF254 (Qingdao Marine Chemical Industry Factory). Fractions were monitored by TLC and spots were visualized by heating after spraying with 5% H_2_SO_4_ in ethanol.

### 3.2. Plant Material

The whole plant of *P. Sibirica* L. Var *megalopha* Fr. were collected in Yongshan, Yunnan Province, People’s Republic of China. A voucher specimen (HUA002) was identified by Prof. Fan Du (Southwest Forestry University) and was deposited at the College of Forestry, Southwest Forestry University.

### 3.3. Extraction, Isolation and Characterization

The 75% ethanol extract of the whole plant of *Polygala sibirica* L. var *megalopha* Fr. (5.0 kg), was chromatographed over a D_101_ macroporous resin, eluted successively with a gradient of H_2_O, 35% EtOH, 65% EtOH, 95% EtOH,. The 65% EtOH eluate was subjected to silica gel column chromatography and eluted with CHCl_3_–MeOH (50:1→0:1) to afford thirteen fractions (Fr.1→Fr.13). Fr.8 (56 g) was chromatographed on a silica gel column and RP-C18 to yield eleven fractions (Fr.8-1→Fr.8-11). Fr.8-7 (35 mg) was purified by Sephadex LH-20 with CHCl_3_–MeOH (1:1) and semi-preparative HPLC with a mixture of acetonitrile and water (34:66, *v*/*v*) to give compound **1** (20 mg). Fr.8-5 (4.654 g) was repeatedly subjected to silica gel column and Sephadex LH-20 chromatography to yield five fractions (Fr.8-5A→Fr.8-5E). Fr.8-5C (40.8 mg) was purified by semi-preparative HPLC with a mixture of acetonitrile and water (29:71, *v*/*v*) to afford **2** (4.6 mg) and **3** (4.3 mg).

Polygalin H (**1**). Yellow amorphous power; ${\left[\alpha \right]}_{D}^{20}$ −89(*c* 0.0011, MeOH); UV (CH_3_OH) λ_max_ (log ε): 343 (0.17), 273 (0.19), 258 (0.19), 209 (0.45); IR (KBr) ν_max_: 3440, 2926, 1613, 1512, 1484, 1354, 1263, 1213, 1142, 1078, 1024 cm^−1^; for ^1^H- and ^13^C-NMR, see Table 2; MALDI-TOF/TOF-MS: *m*/*z* 691.1077 [M + Na]^+^ (calcd. for 691.1640, C_30_H_36_O_17_Na^+^).

Polygalin I (**2**). Yellow amorphous power; ${\left[\alpha \right]}_{D}^{20}$ −89 (*c* 0.0011, MeOH); UV (CH_3_OH) λ_max_ (log ε): 343 (0.17), 273 (0.19), 258 (0.19), 209 (0.45); IR (KBr) ν_max_: 3441, 2925, 1609, 1510, 1484, 1351, 1258, 1212, 1180, 1077, 1024 cm^−1^; for ^1^H- and ^13^C-NMR, see Table 2; MALDI-TOF/TOF-MS: *m*/*z* 675.1942 [M + Na]^+^ (calcd. for 675.1901, C_30_H_36_O_16_Na^+^).

Polygalin D (**3**). Yellow needles (MeOH); m.p. 251–253 °C; ${\left[\alpha \right]}_{D}^{20}$ −47 (*c* 0.51, MeOH); UV (CH_3_OH) λ_max_ (log ε) nm: 268 (4.12), 355 (3.98); IR (KBr) cm^−1^: 3410, 2943, 1657, 1601, 1497, 1353, 1218 and 1025. HRESIMS (positive) *m*/*z*: 647.1587 (calcd. for C_28_H_32_O_16_, 647.1588]; ^1^H-NMR (DMSO-*d*_6_, 600 MHz) δ_H_: 7.82 (1H, dd, *J* = 9.0, 2.0 Hz, H-6′), 7.59 (1H, d, *J* = 2.0 Hz, H-2′), 7.03 (1H, d, *J* = 9.0 Hz, H-5′), 6.72 (1H, d, *J* = 2.5 Hz, H-8), 6.38 (1H, d, *J* = 2.5 Hz, H-6), 3.88 (3H, s, 4′-OMe), 3.87 (3H, s, 7-OMe), 5.63 (1H, d, *J* = 8.0 Hz ,Glc-H-1); ^13^C-NMR (DMSO-*d*_6_, 150 MHz) δ: 177.8 (s, C-4), 165.1 (s, C-7), 161.0 (s,C-5), 156.2 (s, C-9), 155.7 (s, C-2), 150.1 (s, C-4′), 145.0 (s, C-3′), 133.7 (s, C-3), 122.5 (s, C-6′), 121.8 (s, C-1′), 115.5 (s, C-2′), 111.1 (s, C-5′), 108.6 (d, Api-C-1), 105.0 (s, C-10), 98.5 (d, Glc-C-1), 97.8 (s, C-6), 92.1 (s, C-8), 79.2 (s, Api-C-3), 77.5 (d, Glc-C-2), 77.0 (d, Glc-C-3, 5), 76.1 (d, Api-C-2), 73.9 (t, Api-C-4), 70.2 (d, Glc-C-4), 64.2 (t, Api-C-5), 60.8 (t, Glc-C-6), 56.1 (q, 7-OMe), 55.6 (q, 4′-OMe).

**Table 2:** The ^1^H-NMR and ^13^C-NMR data of **1**, **2** in DMSO-*d*_6_ (600 MHz).

No.	1		2	
	δ_H_, *J* (Hz)	δ_C_	δ_H_, *J* (Hz)	δ_C_
2		155.6 s		155.4 s
3		133.9 s		133.8 s
4		177.7 s		177.7 s
5		159.3 s		159.3 s
6		108.0 s		108.0 s
7		163.9 s		163.9 s
8	6.81 (s)	90.3 d	6.86 (s)	90.4 d
9		156.1 s		156.1 s
10		104.5 s		104.6 s
11	4.39 (s)	60.8 t	4.39 (s)	60.8 t
1′		122.5 s		122.4 s
2′	7.58 (s)	115.3 d	8.27 (d, 8.5)	130.9 d
3′		146.1 s	7.03 (d, 8.5)	113.7 d
4′		150.2 s		161.3 s
5′	6.97 (d, 8.7)	111.2 d	7.03 (d, 8.5)	113.7 d
6′	7.94 (d, 8.7)	122.2 d	8.27 (d, 8.5)	130.9 d
5-OMe	3.21 (s)	57.3 q	3.22 (s)	57.3 q
7-OMe	3.91 (s)	56.5 q	3.91 (s)	56.5 q
4′-OMe	3.87 (s)	55.6 q	3.85 (s)	55.4 q
Gal-1″	5.62 (d, 7.7)	98.9 d	5.61 (d, 7.6)	98.9 d
2″	3.76 (m)	74.9 d	3.76 (m)	74.9 d
3″	3.57 (m)	73.8 d	3.83 (m)	73.7 d
4″	3.63 (s)	68.3 d	3.63 (m)	68.2 d
5″	3.33 (m)	75.8 d	3.34 (m)	75.7 d
6″	3.42 (m)	60.0 t	3.41 (m)	60.0 t
	3.27 (m)		3.25 (m)	
Api-1‴	5.31 (s)	108.8 d	5.31 (s)	108.8 d
2‴	3.80 (m)	76.1d	3.81 (m)	76.1 d
3‴		79.2 s		79.2 s
4‴	3.83 (d, 9.2)	73.9 t	3.84 (m)	73.8 t
	3.49 (d, 9.0)		3.57 (m)	
5‴	3.42 (m)	64.2 t	3.41 (m)	64.2 t
	3.37 (m)		3.34 (m)	

### 3.4. Acid Hydrolysis of Compound ***1***

A solution of **1** (4.8 mg) in 1 M HCl (3 mL) was heated in a water bath at 70 °C for 6 h. After cooling, the reaction mixture was neutralized with NaHCO_3_ and extracted with CHCl3. Through TLC comparison with an authentic sample using EtOAc–EtOH–H2O (5:2:1) as a developing system, galactose and apiose were detected in the water layer (R_f_ = 0.35 and 0.48, respectively). The aqueous solution was further concentrated to dryness and dissolved in 1 mL pyridine. Then hydroxylammonium hydrochloride (1 mg) was added. The solution were kept in a 80 °C water bath for 30 min, followed by treatment with 1 mL Ac_2_O and kept in a 90 °C water bath for another 40 min. The authentic monosaccharide samples were treated similarly as the hydrolysis products. Finally, 1 μL of these acetylated sugars were injected into a HP-20M capillary column using N_2_ as carrier (oven temp. 210 °C) to be analyzed by GC using a FID detector (detector temp. 280 °C). Ultimately, the galactose and apiose were confirmed to be both d-configuration by comparing the retention times with those of standard samples: t_R_: d-galactose 12.086 min, d-apiose 4.450 min, respectively.

### 3.5. Bioassay of Xanthine Oxidase Inhibitory Activity

The enzyme xanthine oxidase catalyses the oxidation of xanthine to uric acid. Inhibition of xanthine oxidase results in a decreased production of uric acid. The uric acid production was measured according to the increasing absorbance at 290 nm. Test solutions were prepared by adding xanthine (final concentrations 50 μM), hydroxylamine (final concentration 0.2 mM), EDTA (final concentration 0.1 mM), and flavonol glycosides in four concentrations (2.5, 5.0, 10.0, 20.0 μM). The reaction was started by adding 0.2 mL of xanthine oxidase (6.25 mU/mL) in a phosphate buffer solution (pH = 7.50, 200 mM). The mixture (total 1 mL) was incubated for 30 min at 37 °C. Prior to the measurement of uric acid prodution, the reaction was stopped by adding 0.1 mL of HCl (0.58 M) [7]. Allopurinol (2.5, 5.0, 10.0, 20.0 μM), a known xanthine oxidase inhibitor, was used as a positive control. The IC_50_ values were calculated by the mean data values from three determinations.

## 4. Conclusions

The chemical investigation of *P. sibirica* L. var *megalopha* Fr. lead to the isolation of two new polygalin H (**1**) and polygalin I (**2**), as well as one known compound, polygalin D (**3**). The bioassay *in vitro* indicated that polygalin D had a high inhibitory activity against xanthine oxidase with an IC_50_ of 16.00 μM. Moreover, those two new flavonol glycosides with rare hydroxymethyl function at C-6 in the skeleton of flavonoids showed stronger inhibitory activities against xanthine oxidase, with an IC_50_ of 9.48 μM for **1** and 8.31 μM for **2**, which deserved to be further studied.
